# Proteotranscriptomic Analysis and Toxicity Assay Suggest the Functional Distinction between Venom Gland Chambers in Twin-Spotted Assassin Bug, *Platymeris biguttatus*

**DOI:** 10.3390/biology11030464

**Published:** 2022-03-17

**Authors:** Fanding Gao, Li Tian, Xinyu Li, Yinqiao Zhang, Tianfang Wang, Ling Ma, Fan Song, Wanzhi Cai, Hu Li

**Affiliations:** 1 Department of Entomology and MOA Key Lab of Pest Monitoring and Green Management, College of Plant Protection, China Agricultural University, Beijing 100193, China; ringgfd2018@cau.edu.cn (F.G.); ltian@cau.edu.cn (L.T.); lixinyu91@cau.edu.cn (X.L.); zhangyinqiao@cau.edu.cn (Y.Z.); malingpotato@163.com (L.M.); fansong@cau.edu.cn (F.S.); caiwz@cau.edu.cn (W.C.); 2 Genecology Research Centre, University of the Sunshine Coast, Maroochydore, QLD 4558, Australia; twang@usc.edu.au

**Keywords:** *Platymeris* *biguttatus*, venom gland, transcriptome, proteome, toxicity assay

## Abstract

**Simple Summary:**

Assassin bugs are one of the diversified groups of venomous predatory insects. Their venom is secreted from three different lumens of the salivary gland and can lead to paralysis, lethality, and liquidation of the prey, respectively. Nevertheless, most reduviid venom components responsible for these effects are not clear. In this study, we use transcriptomics and proteomics to determine the effective salivary protein components in the separate lumen of salivary gland from twin-spotted assassin bug *Platymeris biguttatus* and to conduct toxicological analysis on the function of the salivary gland compartments. Our study sheds light on the functional cooperation between different salivary gland lumens of assassin bugs and will further the understanding of physiological adaptations of venom-based predation and defense in venomous hemipterans.

**Abstract:**

Assassin bugs use their salivary venoms for various purposes, including defense, prey paralyzation, and extra-oral digestion, but the mechanisms underlying the functional complexity of the venom remain largely unclear. Since venom glands are composed of several chambers, it is suggested that individual chambers may be specialized to produce chemically distinct venoms to exert different functions. The current study assesses this hypothesis by performing toxicity assays and transcriptomic and proteomic analysis on components from three major venom gland chambers including the anterior main gland (AMG), the posterior main gland (PMG), and the accessory gland (AG) of the assassin bug *Platymeris biguttatus*. Proteotranscriptomic analysis reveals that AMG and PMG extracts are rich in hemolytic proteins and serine proteases, respectively, whereas transferrin and apolipophorin are dominant in the AG. Toxicity assays reveal that secretions from different gland chambers have distinct effects on the prey, with that from AG compromising prey mobility, that from PMG causing prey death and liquifying the corpse, and that from AMG showing no significant physiological effects. Our study reveals a functional cooperation among venom gland chambers of assassin bugs and provides new insights into physiological adaptations to venom-based predation and defense in venomous predatory bugs.

## 1. Introduction

Venoms are vital to the ecological success of some animals by assisting many important life activities, including prey hunting, defense, and conspecific competition [1,2]. Some animals produce venoms with distinct functions depending on the environmental challenges they confront. For instance, cone snails *Conus geographus* can generate two distinct venoms for defense and predation, respectively, with the venom used for defense containing unique, paralytic toxins [3]. Two gene families from the venom of the giant red bull ant *Myrmecia gulosa*, were found to produce pain in mammals and incapacitate arthropods [4]. Scorpion *Parabuthus transvaalicus* can secrete transparent or cloudy venom with different physical properties depending on the stimulations [5]. Although the functional complexity of venom provides an intriguing case of evolutionary adaptations, its underpinning mechanism remains largely unknown and has only been studied in a handful of species [6].

Assassin bugs (Hemiptera: Reduviidae) use their salivary venom to tackle different environmental challenges, including defense [7,8], prey paralyzation, and extra-oral digestion (EOD hereafter) [9,10,11], as well as blood-feeding [12]. Thus, these bugs provide an ideal model to study molecular mechanisms underlying the evolution and maintenance of the functional diversity of animal venom. The venom gland of assassin bugs is a complex organ consisting of three pairs of separate chambers, including two anterior main glands (AMGs), two posterior main glands (PMGs), and two accessory glands (AGs). Venom produced by these gland chambers can engage either in predation by killing the prey and breaking down prey tissue (during EOD) or in defense by causing a pain reaction to bird or mammal predators [13,14]. Previous researchers have speculated that the multifunctional venom is a result of functional specialization of these gland compartments, with each producing a chemically distinct venomous secretion to exert a distinct function. Previous studies have mostly focused on AMG and PMG, and empirical studies on different species have yielded inconsistent results. For instance, through toxicity assays, Haridass et al. [15] studied the extracts of the different gland compartments of three assassin bug species and found that the AMG of these species were the primary source of venom used to paralyze the prey, and PMG secreted enzymes for the EOD. Combining the toxicity assay and proteomic and transcriptomic analysis, Walker et al. [13] analyzed the roles of AMG and PMG extracts of *Pristhesancus plagipennis* (Reduviidae: Harpactorinae) during predation and defense. The study revealed that extracts of these chambers contain different sets of proteins and peptides. The toxicity assay on the separate gland chamber extract showed that only PMG extract can paralyze the prey. A subsequent similar study on *Platymeris rhadamanthus* revealed that the PMG venom not only kills the prey but also may potentially cause pain to mammalian predators [7], suggesting that PMG carries out the dual function of prey manipulation and defense. Fischer et al. [14] studied the involvement of AMG and PMG venom chambers during the defensive response to different degrees of artificial disturbance in *Pl. biguttatus* and *Psytalla horrida*. The study found that in *Pl. biguttatus*, PMG is the only chamber used for defense, whereas in *Ps. horrida*, both AMG and PMG secretion participate in defense. However, in *Ps. horrida*, the extent of involvement of these two chambers is context-dependent, with PMG responding to minor harassment and AMG to intensive harassment. Although the highly complex function of the main venom gland chambers from assassin bugs has been studied across several species [7,13,14,15], the function of AG remains largely unexploited.

In contrast to AMG and PMG, our knowledge about the components and toxicity of AG is relatively limited in assassin bugs. The reasons for this are partially the AG dissection difficulty resulted from its structural fragility and the relatively low concentration of the AG secretion [13]. A few empirical studies on AG suggested that this chamber plays a minor role in defense and predation but serves primarily as a water reservoir. For instance, using a toxicity assay, the role of AG during predation in *Pl. rhadamanthus* was studied, which found that the injection of AG extract has no effects on the American cockroach, *Periplaneta americana* [9]. In 1981, Haridass et al. [15] studied the function of salivary gland of three assassin bugs, *Haematorrhophus nigroviolaceus* (Ectrichodiinae), *Pirates affinis* (Peiratinae), and *Triatoma rubrofasciata* (Triatominae), conducted the injection experiment on the beetle *Omphora pilosa*, found AG has no significant effects on the immobilization or EOD, and suggested AG was the production of water saliva. Although later generations have discovered the existence of proteins in AG in the relative studies of the salivary gland [13,16,17], the exact functions of these protein venoms are unclear.

In the current study, we characterize the function of AMG, PMG, and AG in *Pl. biguttatus* using proteotranscriptomics and toxicity assays. We first provide a detailed morphological characterization of these chambers in the form of high-resolution images of the venom glands. We then assess the toxicological effects of chamber-specific glandular extract on putative prey insects. To gain further insights into the functional specialization of each chamber at molecular level, we perform transcriptomic and proteomic analysis to examine the protein components of the three glandular extracts. Our study suggests that the three venom gland chambers of *Pl. biguttatus* play distinct functions in venom production and discovered that AG may also play a role in predation, which has not been recognized by previous research and merits further study. Overall, our study sheds light on the molecular mechanisms underlying venom multifunctionality, physiological adaptations to venom production, and usage in some assassin bugs.

## 2. Materials and Methods

### 2.1. Salivary Venom Sampling by Stimulation Treatments

**Figure 1:**
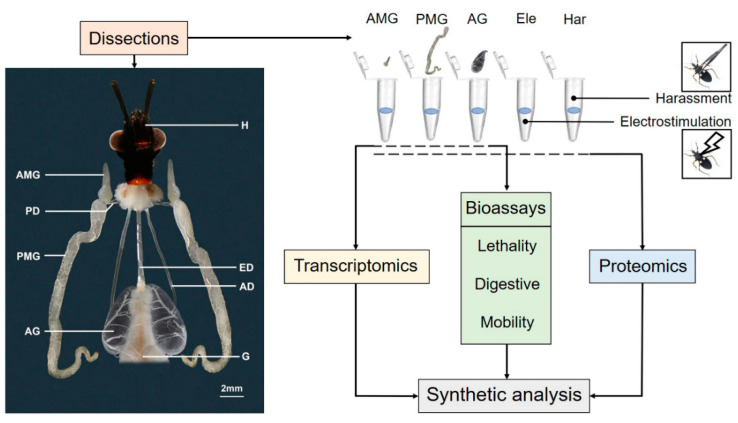
The structure of the salivary gland of *Platymeris biguttatus* in a ventral view. Schematic workflow of combined transcriptomic, proteomic, and toxicological assays of the salivary gland of *Pl. biguttatus*. Abbreviation: AD, accessory duct; AG, accessory gland; AMG, anterior main gland; ED, esophageal duct; Ele, venom of electrostimulation; G, gut; H, head; Har, venom of harassment; PD, principal duct; PMG, posterior main gland.

Adults of *Pl. biguttatus* were reared in the laboratory at room temperature (25 °C). Before treatment, adult assassin bugs were anesthetized by chilling and isolated into individual plastic discs. Following the method described by Walker [13], we treated the assassin bugs with two types of stimulation to induce predatory and defensive responses. To stimulate a predatory response, an electrostimulation (Ele) treatment was applied to the insects after one day of isolation. In general, the thorax muscle was treated using electrodes (30 V, 5 ms pulses, 5 Hz), with YC-2 programmed stimulator (Chengdu Instrumeny Factory, Chengdu, China). Walker [13] has demonstrated that such treatment can stimulate the physiological response of the assassin bug similar to its reaction to a real prey. To stimulate defensive response, we simulate the harassment by a bird or mammal predator by teasing the bug repeatedly with tweezer (Har treatment) [18]. The salivary fluid discharged by assassin bugs in response to Ele and Har treatments were considered venom for predation and defense, respectively. To collect the venom samples, proboscis of the treated assassin bugs was gently inserted into a tube to collect venom fluid. The tube containing saliva was immediately transferred on ice and stored at −80 °C for subsequent experiments (as shown in Figure 1). For each treatment, three biological replicates were performed. The saliva of eight individuals was pooled for each replicate.

### 2.2. Salivary Venom Collection by Venom Gland Dissection

To collect extracts from individual gland chambers directly, we dissected the venom gland and isolated the AMG, PMG, and AG on dry ice. After isolation, the individual compartment was placed in ice-cold phosphate-buffered saline (PBS, pH 7.2) and immediately transferred onto dry ice. To obtain the extracts [13], the tubes were vibrated using a MixStar (DLAB, Beijing, China) for 10 s and centrifuged (5000 rcf for 1 min at 4 °C) to release fluid content and avoid the histiocytic rupture. The gland tissue was then removed, with the raw extracts centrifuged at 17,000 rcf, 4 °C for 10 min, and the supernatants were obtained and stored at −80 °C until further analysis. The concentration of total protein in saliva was measured using BCA Protein Assay Kit (TianGen Biotech, Beijing, China). All the salivary venom collected as above was used for the following transcriptomic, proteomic, and toxicological experiments (Figure 1).

### 2.3. Gland Imaging

Venom glands were dissected from adult assassin bugs in PBS and imaged using a Nikon SMZ18 stereomicroscope equipped with a Canon EOS 7D digital camera (Canon, Tokyo, Japan). The photographs were superimposed using Helicon Focus (Helicon Soft Ltd., Kharkiy, Ukraine) and processed using Adobe Photoshop CC 2019 (Adobe Systems Inc., San Jose, CA, USA).

### 2.4. RNA-SEQ and De Novo Transcriptome Assembly

Adult bugs were dissected on dry ice after anesthetization by chilling, and AMG, PMG, and AG were isolated and snap frozen in liquid nitrogen. Total RNA was extracted using the RNA Miniprep Kit (Qiagen, Hilden, Germany) following kit protocol. Concentrations were measured using NanoDrop2000 (Thermo Scientific, Bremen, Germany). Total amounts of RNA used for transcriptome sequencing were 2.33 µg for AMG, 33.12 µg for PMG, and 2.44 µg for AG. The transcriptomes were sequenced with paired-end reads of 150 bp to obtain 6 Gb data using an Illumina NovaSeq 6000 at Berry Genomics (Beijing, China). Raw reads were trimmed using Fastp [19], and 47,710,096 clean reads of AMG, 38,670,618 clean reads of PMG, and 37,498,542 clean reads of AG were obtained. Sequences of the three glandular compartments were assembled separately from reads by the software Trinity [20] using the default trimming parameters and reduced redundancy by clustering using software CD-HIT-v4.8.1 (sequence identity threshold default 0.9 and word length set to 8) [21]. For further protein identification of LC-MS/MS, all open reading frames (ORFs) that are longer than 90 bp in the final transcriptomes of all compartments were translated via TransDecoder (TransDecoder, RRID: SCR_017647) to generate an amino acids sequences library. Totals of 22,570, 32,881, and 25,038 sequences were predicted, respectively, from AMG, PMG, and AG, and these sequences were annotated based on Uniref90 database by BLASTp (E-value < 10^−5^). The expression of proteins in transcriptome were quantified using RSEM [22] and estimated with TPM (transcripts per million).

### 2.5. NanoLC-MS/MS Analysis and Sequence Annotation of Secreted Proteins

The extracted salivary proteins of each gland, Ele and Har, were incorporated into SDT buffer (4% SDS, 100 mM Tris-HCl, 100 mM dithiothreitol, pH 7.6), and peptides were obtained by digestion with trypsin according to Filter-Aided Sample Preparation (FASP) described by Wiśniewski [23]. For LC-MS/MS, peptides were equivalent using a NanoDrop2000 spectrophotometer at OD 280 and loaded onto Easy nLC Liquid Chromatograph (Thermo Scientific, Bremen, Germany) with a reverse-phase trap column (Thermo Scientific Acclaim PepMap100, nanoViper C18), connected to the C18-reversed-phase analytical column (Thermo Scientific Easy Column) in buffer A (0.1% Formic acid). Peptides were separated with a linear gradient of buffer B (84% acetonitrile and 0.1% Formic acid) at a flow rate of 300 nl/min controlled by IntelliFlow technology (0–55% buffer B for 110 min, 55–100% buffer B for 5 min, held in 100% buffer B for 5 min).

The LC outflow was coupled with a Q Exactive Mass Spectrometer (Thermo Scientific, Bremen, Germany) operated in positive ion mode. MS data were acquired using a data-dependent top10 method dynamically choosing the most abundant precursor ions from the survey scan (300–1800 *m*/*z*) for HCD fragmentation. The automatic gain control (AGC) target was set to 3e6, and the maximum inject time was 10 ms. Dynamic exclusion duration was 40 s. Survey scans were acquired at a resolution of 70,000 at 200 *m*/*z*, resolution for HCD spectra was set to 17,500 at 200 *m*/*z*, and isolation width was 2 *m*/*z*. The normalized collision energy was 30 eV, and the underfill ratio, which specifies the minimum percentage of the target value likely to be reached at maximum fill time, was defined as 0.1%.

To further assess protein components in our salivary and venom gland samples, we searched our mass spectra data against amino acid sequence libraries translated from the glandular transcriptome. In general, mass spectra of individual glandular extractions were compared to the amino acids sequences library translated from its own transcriptome data, respectively, using MaxQuant 1.3.0.5 software [24] (digestion chose Trypsin, and global parameters chose iBAQ). Mass spectrum data of salivary secretions from Har and Ele stimulation were searched against amino acid sequences translated from all three glandular transcriptomes. The relative quantity of each protein in individual salivary or glandular samples was quantified using the iBAQ value (normalized to intensity-based absolute quantification of proteins) [25] given by MaxQuant.

With predicted protein structure, we screened venom proteins from the proteome data based on several criteria: (1) possession of a signal peptide predicted by SignalP 5.0 [26], (2) lacking a retention signal endoplasmic reticulum (ER) C-terminal KDEL or HDEL sequence [27], (3) lacking transmembrane regions or on the signal peptide predicted by TMHMM [28], and (4) localized to extracellular space identified by BUSCA [29].

For protein annotation, we constructed a database named ReduSGP (Supplementary Data S1) containing all previously annotated secreted proteins of Reduviidae [7,13,14]. The filtered secreted proteins were annotated based on Uniref90 and ReduSGP databases by BLASTp (E < 10^−5^) and classified based on Pfam and InterPro databases by InterProScan [30]. For proteins without any blast hit, we built another database of shortlisted secreted protein from *Pl. biguttatus* and BLASTp homology (E < 0.01). Unannotated proteins were categorized based on their homology and were all named putative reduvenom family protein.

### 2.6. Toxicological Bioassays of Salivary Glands

To investigate the lethal effects of different salivary glands on prey insects, AMG, AG, PMG extracts, and Ele were injected into the left second proleg (see Supplementary Figure S1A) of the great wax moth *Galleria mellonella* larvae using a Nanoject III microsyringe pump (Drummond Scientific, Broomall, PA, USA). Since our toxicity assay showed that PMG and Ele had lethal effects on *G. mellonella* larvaae, we measured the median lethal dose (LD_50_) and maximum lethal dose (LD_99_) of PMG and Ele extract. We, respectively, injected 5 μL [14] of PMG extracts and Ele with the concentrations gradient (0.4, 0.8, 1.2, 1.6, and 2.0 μg/μL), and 5 μL of phosphate-buffered saline (PBS) and bovine serum albumin (BSA) were injected as the negative control. Seven larvae were injected for each group, and all the experiments were carried out in three biological replicates. Values of LD_50_ and LD_99_ were calculated with a probit model using SPSS Statistics 26 (IBM, Chicago, IL, USA) [31]. The death of larvae was determined based on the observation that larvae that were completely unresponsive to a tweezer touch.

To test the ability of each salivary gland on tissue EOD, *G. mellonella* larvae were injected with 5 μL of separated gland extracts, which were diluted to the same dose based on the protein content of PMG LD_99_ (31.8 μg per larvae), and control groups received three treatments, including killing by freezing, BSA injection, and a real attack by *Pl. biguttatus*. Larvae were dissected and observed after 5, 10, 20, and 30 min after treatment (see Supplementary Figure S1A).

To investigate the effects of different extracts on the mobility of the prey, we injected *G. mellonella* larvae with the same measure of gland extract as above, while using 5 μL of PBS and BSA injections as the negative control. The movement trajectory of treated insects was recorded using a JVC, GC-PX100 vidicon (JVC, Yokohama, Japan), and analyzed with the center of the body as the observation point (see Supplementary Figure S1B). Seven larvae were injected in each group, and all the experiments were carried out in three biological replicates. Statistical analysis was performed using the one-way ANOVA method by OriginPro 9.1 (OriginLab Corporation, Northamptom, MA, USA) [32], with *p* < 0.05 considered significant.

## 3. Results

### 3.1. Morphology of the Salivary Gland

Light microscopy examination reveals that the salivary gland of *Pl. biguttatus* is a complex organ with three anatomically separate gland lumens (as shown in Figure 1), consistent with previous observations in Reduviidae [15,33]. The AMG was short and located on either side of the forepart of the thorax. The PMG was close to the AMG, extended to the abdomen, reaching the third or fourth abdominal segment depending on different individuals. The AG was relatively big and packed with branched ducts. Paired vesicular AG was located on either side of the midgut in the abdominal cavity, and the AG and gut were adjacent but not connected. Besides that, three long ducts were aroused and extended from the mid-region of the AG forwards into the head, uniting to form a common salivary duct.

### 3.2. The Compositions of Secreted Protein in Different Glands

**Figure 2:**
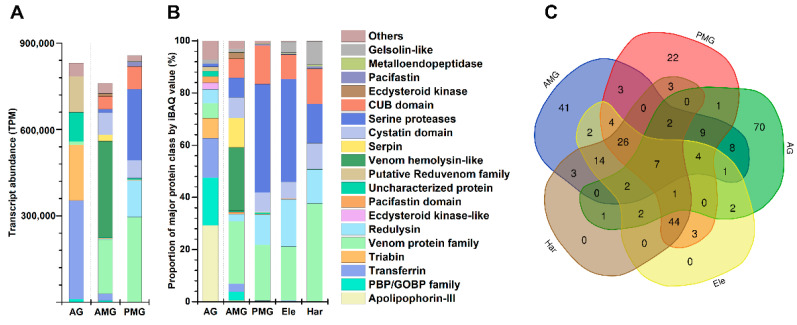
Secreted protein compositions of AMG, PMG, and AG of *Platymeris biguttatus*. (**A**) Transcript abundance of different classes of secreted proteins (normalized to transcripts per million, TPM) of each gland extract; (**B**) proportion of major classes of secreted proteins (normalized to intensity-based absolute quantification of proteins, iBAQ value) in each salivary sample; (**C**) a Venn diagram of protein components among the three glands, Ele, and Har. AG: accessory gland; AMG: anterior main gland; PMG: posterior main gland; Ele: electrostimulation; Har: harassment.

There were 458, 368, and 475 possible secreted proteins predicted from the transcriptomes (TPM > 3) of the glandular lobes of AMG, PMG, and AG, respectively. In AMG, the two dominant proteins were venom family 8-like peptide Pr8a (TPM 149437.6) and venom hemolysin-like protein 2 (TPM 143250.5). In PMG, venom protein family 1 protein 1 (TPM 98782.8) and venom redulysin 2 (TPM 76012.5) were highly expressed. In AG, transferrin (TPM 344146.7) and venom triabin 1 (TPM 143997.7) were the two dominant proteins. Furthermore, secreted proteins were classified into the protein family level based on the protein domain database (Figure 2A).

Among the results, seven proteins in the AMG were categorized into the group of venom hemolysin-like (total TPM 334907.9), followed by the venom protein family (TPM 187617.31, containing 17 proteins) and cystatin domain (TPM 77736.2, four proteins). In the PMG, the identified and categorized with relatively high content were venom protein family (TPM 295686.14, 20 proteins), serine proteases (TPM 246613.5, 50 proteins), and redulysin (TPM 128013.6, six proteins). In the AG, the transferrin-like domain (TPM 344146.74, 1 protein) was the predominant protein family, followed by the putative reduvenom family (TPM 226581.2, including 27 proteins) and redulysin (TPM 192890.4, 2 proteins).

To figure out which glands were mainly responsible for defense and/or predation, we compared the protein contents of AG, AMG, and PMG extracts to the venoms collected by Har and Ele (see Supplementary Table S1). According to Walker et al. (2018), extracts collected via harassment were considered defensive venom, while extracts obtained via electrostimulation were defined as predation and EOD. A total of 234 secreted proteins were identified from the AG, AMG, and PMG extracts, venom from Ele, and venom from Har. There were 111, 130, 130, 116, and 107 proteins identified separately among these five different groups (see Supplementary Table S2). In the AMG, venom hemolysin-like (24.3%), venom protein family (24%), and serpin (11.1%) were the three most abundant classes. In the PMG, the three most abundant protein classes were serine proteases (41.4%), venom protein family (21.4%), and CUB domain (14.8%). Apolipophorin-III (apoLp-III) (29.2%), PBP/GOBP family (18.3%), and transferrin-like domain (15.1%) were the three most abundant classes in the AG. Serine proteases (39.5%), venom protein family (20.7%), and redulysin (18.1%) were the three most abundant proteins in the venom from Ele. In the Har group, the venom protein family (37.4%), serine proteases (15%), and CUB domain (13.5%) were the top three proteins. The protein families in the Ele and Har were highly similar, and both were similar to those in the PMG extracts (Figure 2B). The protein families highly expressed in Ele and Har, such as serine proteases and redulysin, were detected in both AMG and AG, but the contents are lower. Among the proteins obtained by Ele, two proteins (venom cystatin domain peptide Pr15a and AAEL000551-PA) were unique in AMG, three proteins (venom protein family 2 protein 15, venom protein family 6 protein 1, and venom acid phosphatase acph-1-like protein isoform x2) were unique in PMG, and two proteins (alpha-mannosidase and venom pacifastin-like protein 2) were unique in AG. Among the protein obtained by harassment, three proteins (venom CUB domain protein 2, secreted C13 protease-like protein, and venom hemolysin-like protein 2) are unique to PMG, three proteins (venom hemolysin-like protein 7, venom hemolysin-like protein 3, and odorant-binding protein 26) are unique to AMG, and one protein is unique to AG (venom protein family 12 protein 1b (belong to apolipophorin-III)). To further investigate the association of proteins with different contexts of distinct gland compartments, we compared the protein abundance (calculated from iBAQ value) and the expression level (normalized to TPM) detected in the venom samples obtained by two manners with each gland extracts. The venom collected by Ele or Har showed a high correlation with the expressions in both AMG and PMG (see Supplementary Figure S2). The expressions in AG were more correlated with the protein abundance in Ele compared to Har. Overall, the protein analysis assay demonstrated that the extracts of different venom chambers contain distinct sets of proteins and peptides. Each chamber contributes differently to venom produced under different contexts (predation or defense).

### 3.3. Effects of Different Venom Gland Chamber Extracts on Galleria mellonella Larvae

**Figure 3:**
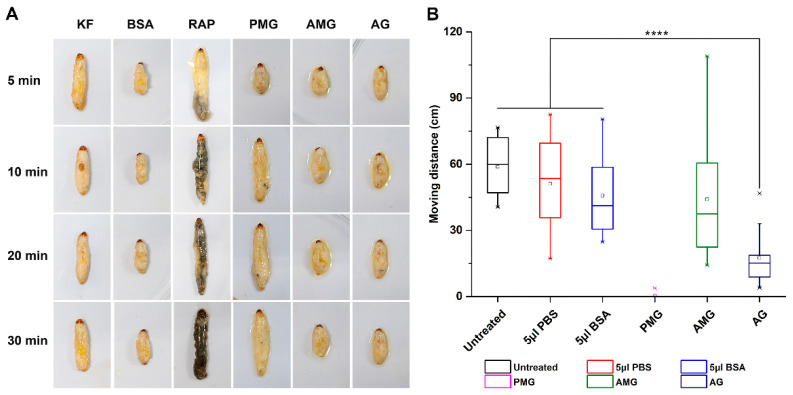
The bioassays of three salivary glands on *Galleria mellonella* larvae. (**A**) Digestive effects of each gland extract on *G. mellonella* larvae at different time points after injections; (**B**) the mobility activities of *G. mellonella* larvae after injections in 10 min. All the images are with the same magnification. AG: injection of accessory gland extracts; AMG: injection of anterior main gland extracts; BSA: injection of bovine serum albumin; KF: killing by freezing; PBS: injection of phosphate buffered saline. PMG: injection of posterior main gland extracts; RAP: the real attack by *Platymeris biguttatus*; **** indicates significant differences among different treatments at *p*-value < 0.0001 (analyzed via one-way ANOVA).

*Galleria mellonella* larvae died quickly after being pierced by *Pl. biguttatus*. The site bitten by *Pl. biguttatus* began darkening after 5 min. Within 30 min after the attack, the corpse turned black and mostly liquified (characterized by the elongated body) (Figure 3A). Similar to the real attack treatment, the PMG extracts also killed most larvae quickly with an estimated LD_50_ of 20.4 μg/g after 30 min, the value was calculated with a probit model by the mortalities (0, 19.05 ± 8.24%, 23.81 ± 16.5%, 42.86 ± 14.29%, and 71.43 ± 14.29% at the concentrations gradient 0.4, 0.8, 1.2, 1.6, and 2.0 μg/μL). The LD_50_ of Ele was 12.5 μg/g computed with the mortalities (0, 23.81 ± 8.24%, 80.95 ± 16.5%, 90.48 ± 8.24%, and 95.24 ± 8.24% at the concentrations gradient 0.4, 0.8, 1.2, 1.6, and 2.0 μg/μL).

PMG injection showed an acute lethal effect on *G. mellonella* larvae. Similar to the real attack treatment, larvae that received PMG injection stopped moving and died immediately, with the corpse liquified within 10 min after treatment. However, different from the real attack treatments, the corpse did not become melanized and remained whitish throughout the 30 minutes’ observation. In contrast, AG and AMG treatment had no lethal effect or obvious tissue breakdown on *G. mellonella* larvae. Larvae treated by the AG extract showed a significant reduction in mobility than the control group (moving distance in 10 min, AG: 17.5 cm (±11.9 cm), and control: 55.9 cm (±21.1 cm), *p*-value < 0.0001) (Figure 3B), whereas the mobility of those received AMG extract was not significantly different from the control.

## 4. Discussion

The compartments in the salivary gland of *Pl. biguttatus* are constituted with symmetric pairs of AMG, PMG, and AG (as shown in Figure 1). Several studies have introduced the morphology of the glandular compartments of the assassin bugs. Haridass et al. [15] recorded the salivary gland morphology of 16 species in Reduviidae by hand drawings. Walker et al. [13] used magnetic resonance imaging and micro-computed tomography to analyze the structure of the venom gland. On the basis of these previous studies, the current study provided high-resolution images of fresh venom glands of *Pl. biguttatus*, which would further enhance our understanding of the anatomical characteristics of venom glands of assassin bugs.

Our study suggested that, similar to other species studied [7,13,14,15], there are indeed functional differentiations among the three pairs of venom gland chambers of *Pl. biguttatus*. We found that the PMG may function to be the primary source of venom used for prey paralyzation and EOD, since there were high contents of redulysin and serine proteases identified. Injection of PMG extraction was similarly lethal to the larvae of the great wax moth as a real attack, suggesting that PMG alone was enough to kill the prey. PMG extract also liquefied the corpse, although it did not cause blackening of the body tissue as the real attack did. This suggests that PMG venom, when being injected alone, can carry out at least partial EOD function. In addition, extracts in response to electrostimulation and harassment, which resemble the environmental stimulation from predation and defense respectively, contain proteins (mainly serine proteases and venom protein family) unique to PMG. Thus, PMG derived venom may engage in dual functions of defense and predation. Supporting the toxicity assay, transcriptomic and proteomic analysis showed that the PMG extract contains abundant serine proteases that belong to family S1 in peptidases (see Supplementary Table S1), a key enzymatic component of assassin bugs venoms that used for EOD [7,34]. Overall, our findings on PMG function are consistent with the results of previous studies [7,13,14].

Although the expression levels of secreted proteins in AMG were highly correlated with the protein abundance in the Ele and Har, our assays did not identify observable toxic function in AMG extract. AMG extract showed no lethal effect on the great wax moth larvae, nor did it compromise the mobility of the treated insects. This is different from the findings reported by Haridass and Ananthakrishnan [15], which implied that AMG is engaged in prey manipulation. However, we detected AMG-specific proteins in both electrostimulation and harassment-induced salivary secretion, which suggested that AMG is likely to contribute to certain venom component(s) used in predation and defense, yet this component(s) might not be bioactive without complexing with venom proteins released by other glands, such as the LD_50_ calculated by the probit model, which requires 20.4 μg/g of PMG and only 12.5 μg/g of Ele, which should be caused by the joint participation of polyglandular compartments. More experiments need to be performed to demonstrate the synergy between glands in the future. Walker et al. [13] suggested that AMG may engage in secreting defensive venom. Supporting this view, our proteomic analysis also identified abundant hemolysin-like protein, a primary functional component in defensive venoms of other venomous insects, in the AMG extract, which is in accordance with Fischer’s finding [14]. Yet, Fischer et al. [14] found no hemolytic effects of AMG extraction on blood agar plates containing human, horse, or sheep erythrocytes. Thus, further behavioral and toxicity assays may be required to clarify the function of these venom proteins produced by AMG.

The previous study on assassin bug venom focused primarily on AMG and PMG [7,13,14], whereas AG has received little attention and been considered not important to venom production. In the current study, it is evident that AG also plays a role in the prey manipulation. In the toxicity assay, the AG extract significantly compromised the mobility of the wax worm. In addition, the AG-specific proteins were detected in both electrostimulation and harassment induced salivary mixtures, suggesting it contributed to venoms used for defense and predation. Although we found that AG extract contains a low abundance of proteins and contains no neurotoxic effects at the level of the molecular function, we detected a considerable amount of redulysin in the AG derived extract. Redulysin was first discovered in triatomine bugs and named trialysin. This protein is a pore-forming lytic protein that induces protozoan lysis and mammalian cell permeabilization, therefore facilitating insect blood-feeding by interfering with host cellular responses [35,36]. Homologous protein is also detected in other assassin bugs and renamed redulysin by Walker [7]. The cell inhibition or lysis activity of the protein may explain the negative effects of AG extract on prey mobility. However, the exact cause of compromised mobility and the role of redulysin remains to be studied by future research. In addition, we found that one of the most abundant proteins in the AG extract is transferrin; the main function of transferrin is iron transportation [37]. Transferrin could have a chelation reaction with different metal ions [38], through which it is known to compromise bacterial survival [39] Thus, a large amount of transferrin in AG suggested that AG secretion may have antibacterial property, which may serve to keep the assassin bug from infection by bacterial pathogens from the captured prey during feeding. The apolipophorin-III was another dominant protein in the AG, which was in a much lower concentration in the AMG and not detected in the PMG. Apolipophorin-III is reported to play an important function in lipid transport and lipoprotein metabolism [40] and associated with immune response by stimulating the production of antimicrobial peptides in insects [41,42]. Thus, we speculate that AG may also function to assist prey feeding by providing a large number of ion and lipid transport proteins while regulating immune response as required, which would need to be experimentally validated in the future. Overall, our toxicity assay and proteomic analysis suggested that AG may play a more important role in prey processing rather than just serving as a water reservoir as previously recognized [15,43].

## 5. Conclusions

In the current study, we conducted a comprehensive functional analysis of the three pairs of venom gland chambers in *Pl. biguttatus* through toxicity assays in conjunction with transcriptomic and proteomic analysis. We further confirmed previous findings in assassin bugs that different venom chambers secrete distinct sets of proteins and engage in venom production to a different extent under different contexts. Therefore, functional specialization of venom gland chambers may be the key mechanism through which assassin bugs produce multifunctional venoms, including serine proteases, apolipophorin-III, and the venom protein family. In addition, we discovered the function of AG during prey manipulation that has not been discovered by previous studies. In *Pl. biguttatus*, the extract from AG may aid predation by compromising prey mobility and assisting EOD and food absorption by providing a large amount of venom fluid that is rich in proteins potentially associated with transport and/or immune response. Overall, our studies suggest assassin bug venom is molecularly complex and there is complicated functional cooperation among venom gland chambers for venom production. Further study is needed to assess the key functional proteins in the venom, to further understand mechanisms regulating functional specialization of venom gland chambers.

## Data Availability

The Illumina sequenced reads have been deposited into NCBI SRA database (accession number: PRJNA801928). The mass spectrometry proteomics data have been deposited to the ProteomeXchange Consortium (http://proteomecentral.proteomexchange.org, accessed on 6 February 2022) under the identifier number PXD031473.

## Supplementary Materials

The following supporting information can be downloaded at: https://www.mdpi.com/article/10.3390/biology11030464/s1, Data S1: A self-built database by newly published documents of secreted protein from related species in Reduviidae, Figure S1: Toxicological bioassay experiments on larvae of *Galleria mellonella* larvae using the extracts from salivary glands, Figure S2: Comparison of protein abundance and expression level in each gland compartment, Table S1: The identification and quantification results of protein and peptides detected by MaxQuant with mass spectra, Table S2: Salivary proteins of adult *Platymeris biguttatus* identified by mass spectrometry and transcriptomic sequencing.
