# Supplementary material for: Proteotranscriptomic Analysis and Toxicity Assay Suggest the Functional Distinction between Venom Gland Chambers in Twin-Spotted Assassin Bug, Platymeris biguttatus
Source: Biology (Basel). 2022 Mar 17;11(3):464. doi: 10.3390/biology11030464 (PMC8945326; doi:10.3390/biology11030464)
Supplement: Supplementary file 1 [file biology-11-00464-s001.zip › Supplementary Figures.pdf]

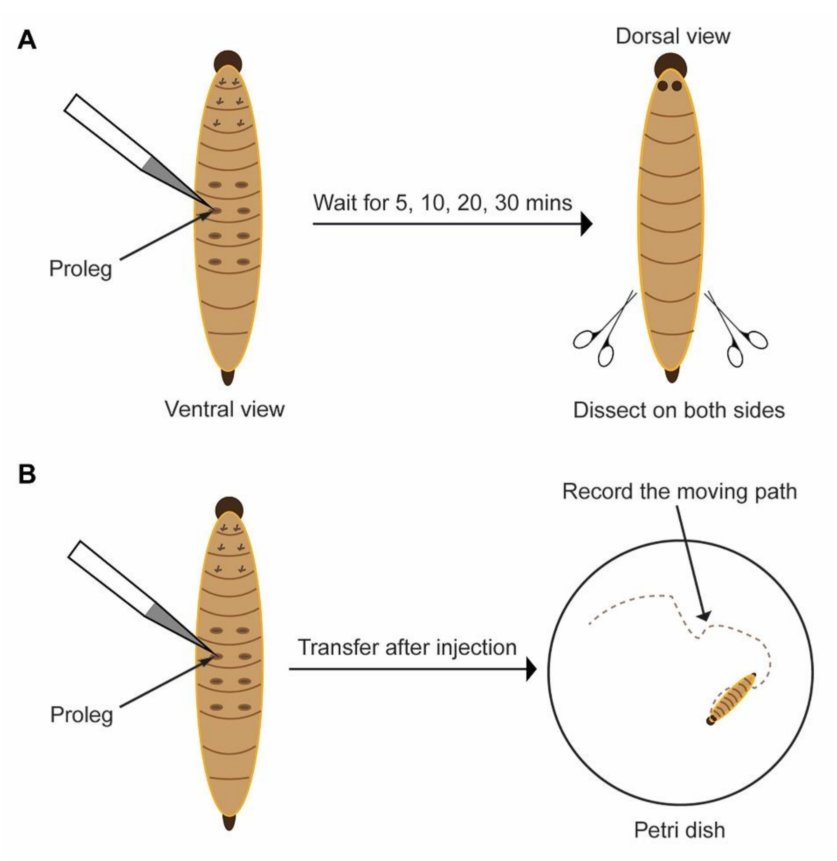


**Figure S1.** Toxicological bioassay experiments on larvae of *Galleria mellonella* using the extracts from salivary glands. (**A**) Evaluation of effects on tissue digestion by venom injection and larval dissection. (**B**) Evaluation of effects on larval mobility by venom injection and moving path record.

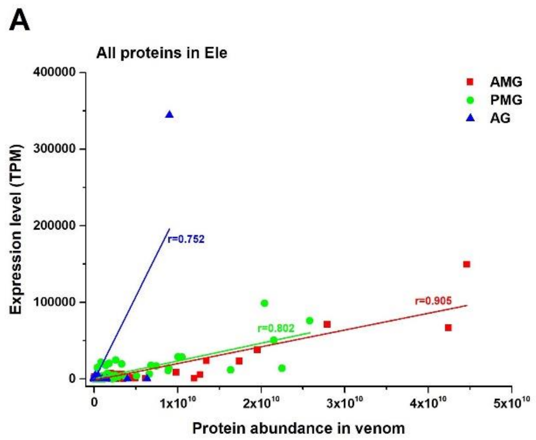


**Figure S2.** Comparison of secretory protein abundance (calculated from intensity-based absolute quantification of proteins, iBAQ value) and expression level (normalized to transcripts per million, TPM) in each gland compartment. (**A**) The identified secretory proteins from venom obtained by electrostimulation. (**B**) The identified secretory proteins from venom obtained by harassment. AG: accessory gland; AMG: anterior main gland; PMG: posterior main gland. Values *r* indicate Pearson correlation coefficient.
